# Relativistic Surface Wave Oscillator in Y-Band with Large Oversized Structures Modulated by Dual Reflectors

**DOI:** 10.1038/s41598-019-55525-9

**Published:** 2020-01-15

**Authors:** Shuang Li, Jianguo Wang, Dongyang Wang

**Affiliations:** 1 Science and Technology on High Power Microwave Laboratory, Northwest Institute of Nuclear Technology, P. O. Box 69-1, Xi’an, 710024 China; 2 Key Laboratory for Physical Electronics and Devices of the Ministry of Education, Xi’an Jiaotong University, Xi’an, 710049 China

**Keywords:** Electrical and electronic engineering, Applied physics

## Abstract

To increase the generation efficiency of the terahertz wave in the Y band, the idea of dual-reflector is introduced in the relativistic surface wave oscillator (SWO) with large oversized structures. The dual-reflector and the slow-wave structure (SWS) construct a resonator where the field strength of TM_01_ mode inside is intensively enhanced and then the efficiency is increased. The pre-modulation on electron beam caused by the reflector is also helpful in improving the output power. Meanwhile, the reflector can reduce the loss of negatively going electrons. Through the particle-in-cell (PIC) simulations, the optimized structure is tested to be stable and little power is transmitting back to the diode area. The output power reaches 138 MW in the perfectly electrical conductivity condition and the frequency is 337.7 GHz with a pure spectrum. The device’s efficiency is increased from 10.7% to 16.2%, compared with the device without any reflectors. The performance of device with lossy material is also focused on. In the situation of copper device, the output power is about 41 MW under the same input conditions and the corresponding efficiency is about 4.8%.

## Introduction

In recent years, the terahertz wave has shown great potential in the applications of remote high-resolution imaging, remote detection of radioactive material, deep space research and communications, plasma diagnostic in nuclear fusion, materials research, biomedical diagnostics, high data rate communications, basic biological spectroscopy, chemical spectroscopy, and so on^1–8^. The prospect of terahertz technology has attracted many researchers to devote to developing the high power terahertz generators, especially the vacuum electronic devices (VEDs)^9–13^. And the slow wave devices are an important class of the VEDs. Based on the interaction between the intense electron beam and the slow-wave structure (SWS), the backward wave oscillator (BWO) becomes a remarkable kind of slow wave device with good performance in high output power and compact structure^14,15^. In a BWO, the electrons are in synchronism with −1^st^ spatial harmonic wave. When a slow wave device operates near the upper edge of the transmission band, it becomes a surface wave oscillator (SWO) whose operation point is close to π point. In an SWO, the fundamental wave slows down to the electron velocity^16^. The *Q*-factor in the SWO is relatively large due to the large reflection and small group velocity, and then the start current is usually lowered in this case^17,18^. What’s more, the large coupling impedance in the SWO is significant in obtaining high interaction efficiency. Besides, use of the surface wave is valid in achieving mode selection in the overmoded structure as well. Thus, the SWO attracts more and more attention in generating millimeter and terahertz waves.

However, their structural dimensions decrease rapidly as the working frequency goes up, causing many crucial problems that must be solved, such as the internal breakdown, limitation of the power capacity, and difficulties in manufacturing and assembling of the device, etc. One possible solution is to use the novel planar structure accompanied with modern micromachining technologies^19–25^, while currently forming and transporting a sheet beam with large width-to-thickness ratio are still challenging, especially for the case in which high output power is pursued^6,26^. Another candidate solution is employing the oversized SWS into the cylindrical device to improve the power-handling capability. The size of enlarged SWS is signed as *D*/λ_0_. Here, *D* is the diameter of the rippled cylindrical waveguide in average and λ_0_ is the wavelength for the operating frequency. If the high order mode such as TM_02_ mode is also considered in propagation in SWS, the minimum radius for the waveguide is denoted as $\frac{D}{2}=\frac{{\chi }_{02}c}{{\omega }_{cT{M}_{02}}}$, where *χ*_02_ = 5.52 is the second root of 0^th^ Bessel function, *c* represents the light speed and ${\omega }_{cT{M}_{02}}$ is the critical frequency for the propagating TM_02_ mode. Thus, when there is no TM_02_ mode in the waveguide, the maximum value for *D*/λ_0_ can be deduced as 1.76. Meanwhile, through the method of expanding SWS, the inner surface area is also meaningful in easing the fabrication difficulties^16,27^.

Based on the idea of the oversized SWS, some high power terahertz devices have been investigated. Based on the quasi-optical theory, Ginzburg *et al*. developed the SWOs with one- and two-dimensional periodic structures^26,28,29^. In China, our research group carried out extensive researches on the oversized SWOs operating at the terahertz range, which are driven by the annular relativistic electron beams^30–34^. The typical experimental results indicated that a compact relativistic SWO generated 154 GHz pulse with repetition rate of 10 and power of 2.6 MW^30^, and its improved version could improve the output power to 5 MW at the frequency of 149 GHz^31^. We also developed an oversized SWO above 0.3 THz^35,36^, whose output power was about 2.1 MW with the duration of about 2 ns. The frequency located in the range of 0.319–0.349 THz^37^. In Japan, Gong *et al*. researched the cylindrical SWOs with the electron beam less than 100 kV^38^. The device can operate in the frequency range of 166–173 GHz and 182–200 GHz, with the radiation power of kilowatts. In South Korea, Min *et al*. designed the 0.1–0.5 THz oversized BWO by utilizing an electron beam of 500 kV and 5 kA, and no further experimental results were reported^39^.

Although the oversized slow wave devices are competitive in generating the high power terahertz waves, the generation efficiency is still very low. According to the practices for enhancing the generation efficiency of the high power microwave sources, some methods are very efficient, such as the non-uniform SWS, klystron-like structure, and resonant reflector, etc^40–46^. Nevertheless, not all of these methods are practicable in the terahertz SWO devices. In a terahertz SWO, for instance, the characteristic size of a ripple is around 0.1 mm, which requires the machining precision at the level of micrometer, so the fabrication of SWS is quite difficult. And hence, the complicated non-uniform structures and klystron-like structure are not suitable for the high-frequency structures in the y-band terahertz SWO.

Unlike the non-uniform SWS and klystron-like structure, the resonant reflector is much easier to be fabricated and configured in the SWO, but there are some key issues to be studied. First, there exists many competing electromagnetic modes in the oversized SWS, how to excite the desired mode in the oversized SWS should be studied. Second, how does the dual-reflector affect the distribution of the electromagnetic field in the oversized SWS? And the third, how does the dual-reflector enhance the efficiency of energy transferred from the electron beam to the terahertz wave? In this paper, we study these key issues and reveal the mechanism for enhancing the efficiency of the oversized SWO with the dual-reflector.

## Design Of The High-Frequency Circuit

**Figure 1:**
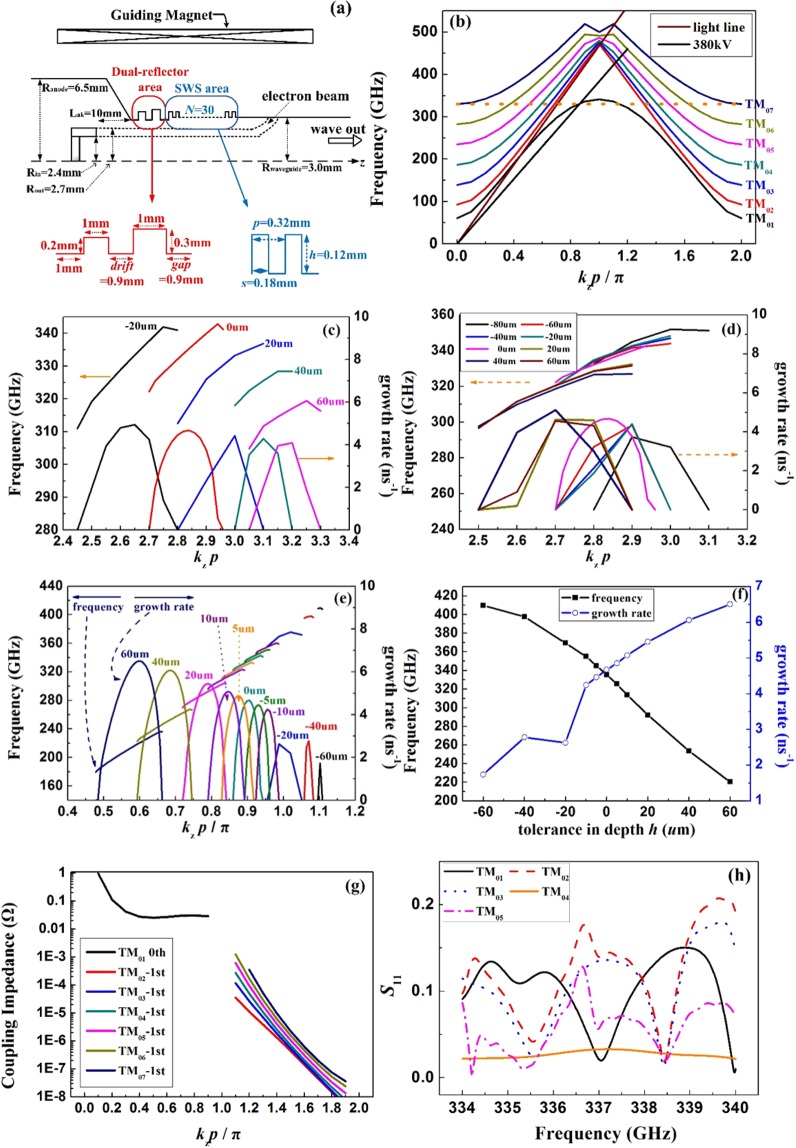
The schematic of the SWO (**a**), the dispersion curves (**b**), the effect of tolerance in periodic length (**c**), the effect of tolerance in width(**d**), the effect of tolerance in depth (**e**,**f**) on the dispersion curves; coupling impedances (**g**) and *S*_11_ parameters (**h**) for the modes in the oversized SWS.

To improve the interaction efficiency, the profile of a SWS that provides a high coupling impedance is preferred^13^. Moreover, considering the difficulty in manufacturing the precise and complex profiles on the metal, the traditional sinusoidal and semicircular shapes are not used. Thus, in this SWO, the circuit of SWS consists of a cylindrical waveguide with rectangular ripples, as shown in Fig. 1(a). Considering the limitation of power capability in the terahertz VED, the oversized SWS is employed here. The averaged radius of SWS is 3 mm, corresponding to the value of *D*/λ_0_ ≈ 6.7^37^.

### Analysis of the dispersion and multi-mode interaction

In this SWO, the electron beam interacts with the fundamental wave and the synchronous point is determined by the synchronous condition of ${v}_{p}\approx {v}_{z}$. *v*_p_ represents the phase velocity for the fundamental wave and *v*_z_ is the electron’s relativistic velocity. Based on the analytical study on the effect of ripple shape on the dispersion^13^, the size of SWS is chosen as *h* = 0.12 mm, *p* = 0.32 mm, and *s* = 0.18 mm. Its dispersive characteristics is depicted in Fig. 1(b), according to Swegle’s analytical theory on the electromagnetic field in SWS, which is convenient to analyze the dispersion in the SWS^47^.

In the terahertz range, the size of ripple is quite small and the tolerances in fabrication may introduce great shift on the device’s performance. So the effect of tolerance on the dispersion is studied to confirm the acceptable tolerance range. As shown in Fig. 1(c), the operating frequency is lowering and the synchronous point is moving to the −1^st^ harmonic wave area as increase of the periodic length. However, the maximum value of growth rate is not seriously affected. So, if the tolerance is restricted in ±20 μm, the operating mode and the growth rate is guaranteed. In Fig. 1(d), the influence of tolerance in width is studied. The operating frequency is gradually decreased as the increase of ripple’s width and the growth rate is slightly enhanced. Clearly, the tolerance in width plays a slight effect on the operating mode and the acceptable tolerance is about ±40 μm. The influence of tolerance in depth is given in Fig. 1(e), indicating serious effect on the frequency and the growth rate. If the value of ripple depth is changed by 60 μm in manufacture, the frequency is shifted around 100 GHz and the operating point is moving far from the π point. With the increase of depth, the value of growth rate and the range of growth rate are both enlarged. Detailed results are shown in Fig. 1(f) and it is obvious that the tolerance in depth should be no more than 10 μm. In summary, in this SWO, the suggested tolerances for the parameters are $h=0.\,{12}_{-0.01}^{0.01}\,{\rm{mm}}$, $p=0.\,{32}_{-0.02}^{0.02}\,{\rm{mm}}$, ${\rm{and}}\,s=0.\,{18}_{-0.04}^{0.04}\,{\rm{mm}}$.

It’s obvious in Fig. 1(b) that the TM_01_ mode is mostly below the light line, while the high order modes are mainly above the light line. Moreover, only the distribution of the wave of TM_01_ mode is shaped as a surface wave with a phase velocity slower than the speed of light, which is valid in enhancing the coupling impedance. Then, the interaction between electron beam and TM_01_ mode is most likely to take place than those of the high order modes. However, in such an oversized structure with large scale, the problem of mode competition is much more complicated. Though the synchronous condition has determined the operation point on the TM_01_ mode for the beam of 380 kV, it is impossible to absolutely prevent the high order modes from interacting with the electron beam. The electron beam interacts with the high order modes at the -1^st^ spatial harmonic wave ranges where the propagating waves are the backward waves. Once the feedback condition is matched, it is possible to excite the high order modes in SWS. Further, the coupling impedances for all the modes are computed in Fig. 1(g). Although the strength for the high order mode is not as high as that for the TM_01_ mode, they take part of the beam’s energy. Based on the viewpoint that we can’t exclude all the high order modes taking part in the interaction process, a possible way to improve the device’s efficiency is to promote the interaction with the designed mode as high as possible. Here the chosen mode is TM_01_ mode. In order to minimize the influence of high order modes on the beam, a feasible method is to enhance the amplitude of TM_01_ mode in the SWS to a predominant level compared to those of the high order modes.

By analyzing the cold characteristic of the SWS cavity consists of thirty ripples, it is clear to find out in Fig. 1(h) that at the designed operating point of 337 GHz, the reflection coefficient for TM_01_ mode is the lowest. The high order modes have high reflection coefficients, meaning that the electric fields for the higher modes are much weaker in the SWS area than that with the TM_01_ mode. So, the resonance characteristics of the optimized SWS are available in differentiating the modes.

### Design of the dual-reflector

Besides optimizing the SWS to increase the reflection coefficient for TM_01_ mode, the adoption of reflector is also useful to enhance the strength of TM_01_ mode in the SWS^43–46^. As mentioned in Section I, the power going back to the diode area is harmful to the cathode and decreases the device’s efficiency^48,49^. Thus, the method of reflector is introduced to minimize the negative going waves and to enhance the pre-modulation on beam to improve the efficiency.

**Figure 2:**
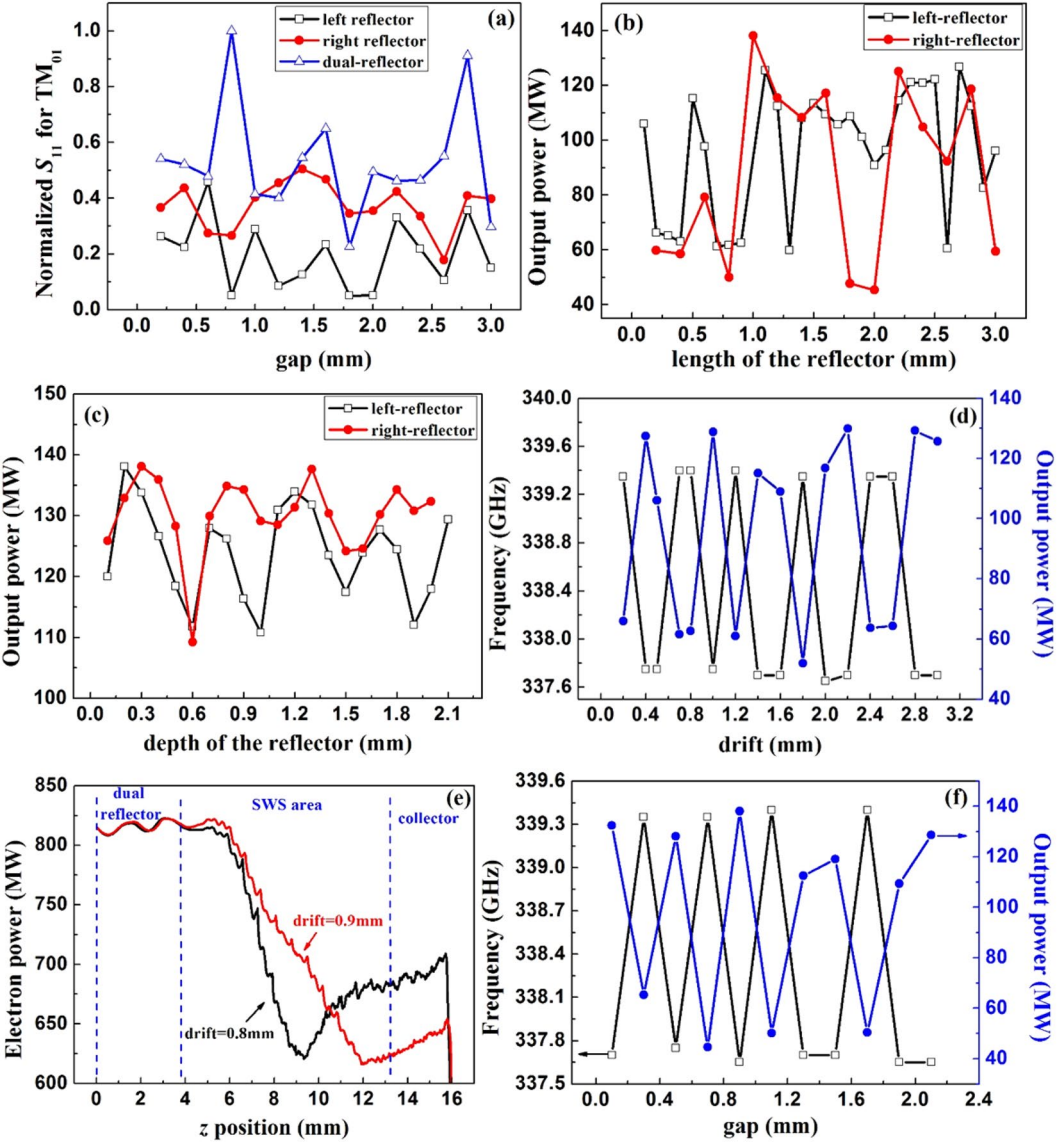
Performance of the reflector system. (**a**) Reflection results for the structures with dual-reflector and one reflector, influence of length(**b**) and depth (**c**) on the output power results in the structure with dual-reflector, influence of drift (**d**–**e**) and gap (**f**) on the output power results in the structure with dual-reflector.

Firstly, the structure of dual-reflector is compared with the traditional case of only one reflector. In Fig. 2(a), the reflection results for the structures with only the left-side reflector, only the right-side reflector and the dual-reflector are compared under various values for the parameter of gap. All the reflection results are normalized to maximum value acquired in the case of dual-reflector with gap = 0.8 mm. It’s clear that the reflection results for the model with two reflectors are much better than that with only one reflector. Thus, a dual-reflector is preferred here to offer sufficient reflection. The configuration of the dual-reflector is schematically shown in Fig. 1(a). The dimensions of the dual-reflector directly affect the modulation on electron beam and the output power, which are shown in Fig. 2(b,c). The influences of its length and depth on the power are approximately periodic. The results of output power are repeated when the value of reflector’s depth is varied as 0.5 mm in Fig. 2(c). This is in accordance with the theoretical analysis on the reflector resonator, whose resonance frequency is defined as ${\omega }_{T{M}_{nm}}=\frac{{\chi }_{nm}}{a\sqrt{\varepsilon \mu }}$, the radius of the reflector resonator operating at certain cut-off mode can be got under the condition of operating frequency as 337.7 GHz. The difference among the radii for the series of resonators with different cut-off modes, such as TM_01_, TM_02_, TM_03_…, is calculated as 0.44 mm, which is quite close to the repetitive space of 0.5 mm in Fig. 2(c). Based on comparison in Fig. 2(b,c), the dimensions of the dual-reflector can be determined and given in Fig. 1(b). In the dual-reflector system, there are two crucial structural parameters, which directly determine the device’s performance, are ‘drift’ and ‘gap’ as labeled in Fig. 1(b). As shown in Fig. 2(d), the parameter ‘drift’ seriously affects the matching between *E*_z_ field and the electron beam. Obviously, the output results are periodically changed with the parameter of drift. In general, the changing period is about 0.6 mm. The frequency results are also periodically affected. When the frequency jumps to 339.4 GHz, the output power reaches the low level. And when the frequency is diagnosed to be 337.7 GHz, the device operates stably and the output power reaches the high level. Obviously, an improper value of drift makes the device apart from the designed status with an unwanted operating mode. So the parameter of drift is selected as 0.9 mm. Moreover, the details of modulations on the electrons are presented in Fig. 2(e), which compares two structures with drift = 0.8 mm and drift = 0.9 mm. With an optimized value of drift = 0.9 mm, the electron power is well extracted at the end of SWS. On the contrary, the situation with an improper value of drift = 0.8 mm is rather different. The electrons absorb the power at the end of SWS area, causing a sharp reduction in output power and unstable device. Similarly, the effects of gap on the output results are periodic in Fig. 2(f). The changing period is about 0.5 mm. When the length of gap is not well matched, the device operates far from the anticipated point of 337.7 GHz. Consequently, the shape and position of the dual-reflector is confirmed based on the above discussions. The values of drift and gap are optimized both as 0.9 mm.

**Figure 3:**
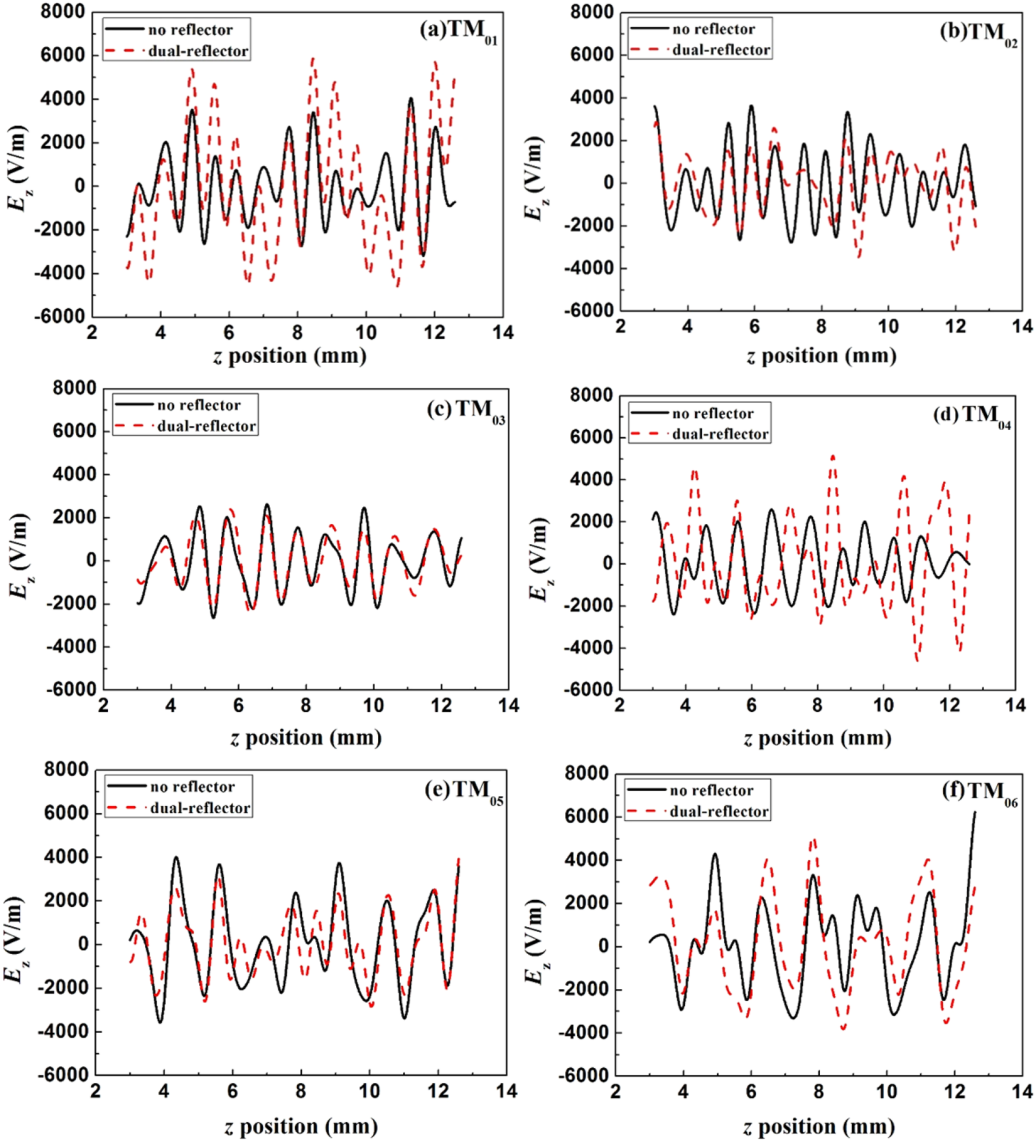
Comparison of the amplitudes for the modes in the structures with and without reflectors.

Based on the optimized dual-reflector, the whole cavity is formed as a perfect resonator, where the amplitudes of *E*_z_ for all modes in the SWS are shown in Fig. 3 and compared at the same position. It is clear that the amplitude of TM_01_ mode is effectively enhanced in the SWS due to the strong reflection from the reflectors. At the same time, the other modes are not obviously enhanced. In fact, the amplitude of the TM_01_ mode is not regnant in the previous structure without reflector [37] and the amplitudes of TM_05_ and TM_06_ are even higher. That is because, when the feedback conditions of the backward waves are reached, the higher modes such as TM_05_ and TM_06_ can be strongly excited in SWS and their amplitudes may be larger than that of TM_01_ mode as well. In that case, the efficiency for the interaction between TM_01_mode and the beam would be seriously disturbed. In contrast, in the case of using reflectors, the situation is greatly improved. The enhanced strength of *E*_z_ for TM_01_ mode is apparently higher than those of the other modes, which is quite meaningful for the improvement on the device’s efficiency and the purification of output signal.

## Particle Simulation Results

**Figure 4:**
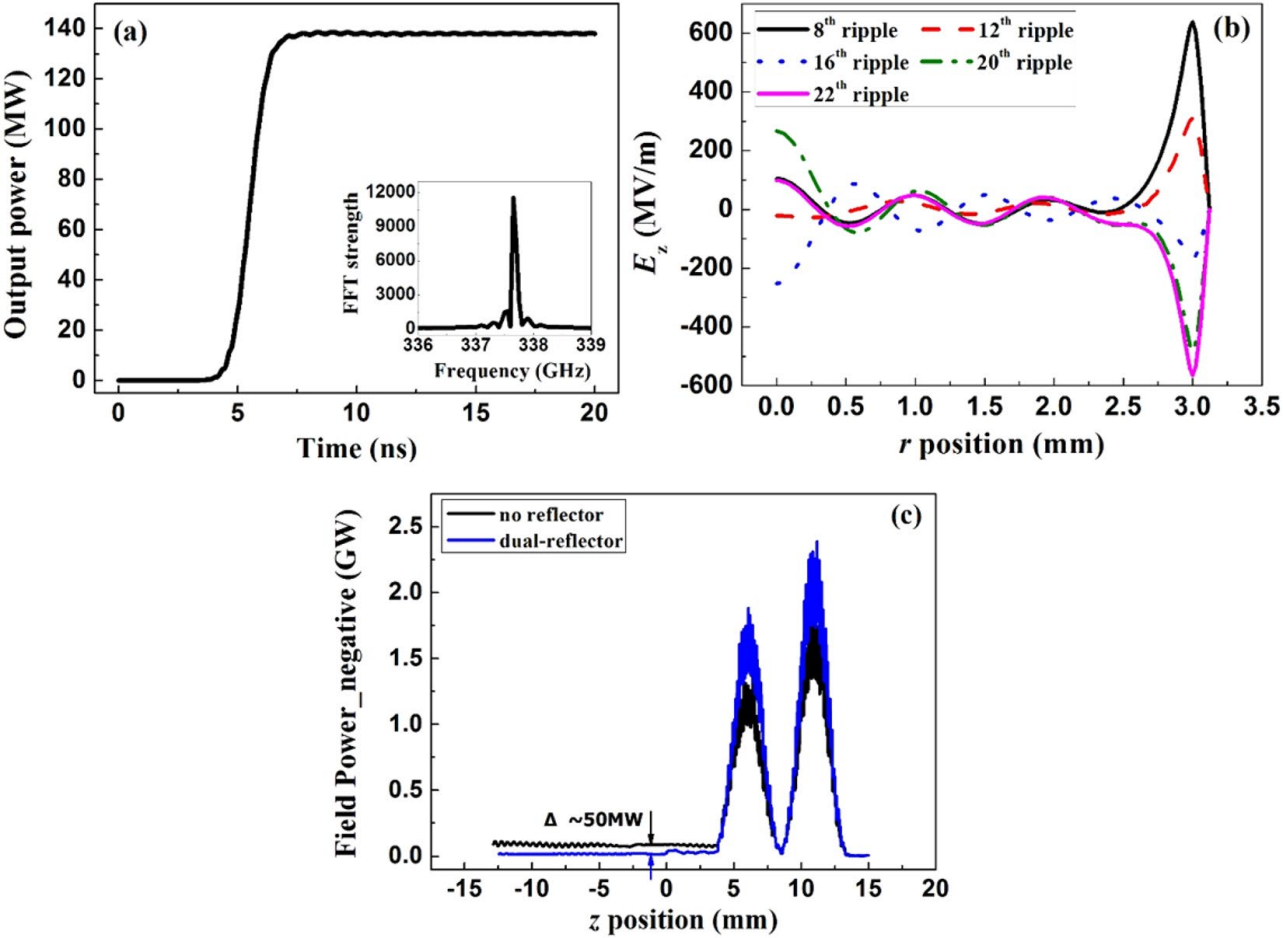
Output results. (**a**) Output power and frequency of terahertz wave; (**b**) *E*_*z*_ vs. *r*; (**c**) field power vs. *z*.

Through the optimization on the SWS and the dual-reflector, the whole SWO configuration is determined. The performance of the optimized structure is studied by the particle-in-cell (PIC) code UNIPIC^50–52^. This code has been used to develop insight into the complex physical mechanisms involved in various VED sources^30,33,41,43,46^. The device is modeled by the two dimensional code and thus only the azimuthally symmetric modes are considered. Actually, as analyzed in [13], the start current of the operating mode TM_01_ is far less than that of the non-symmetric modes in such SWO device. Moreover, in this work, the amplitude of TM_01_ is further enhanced by the dual-reflector. So the TM_01_ mode dominates the interaction process and the non-symmetric modes are comparatively weak. Within the cylindrical coordinate, the computation cell in the model is set as *d*z = *d*r = 0.02 mm. The magnetic field is set as 5 T to guide the electron beam and all the device is immersed in the uniform zone of the magnet. The conditions for the relativistic electron beam are 382 kV and 2.2 kA. The material in simulation is chosen as the perfect conductor (PEC) here^53^, and the lossy material for practical use is considered in the subsequent section. Some results are given in Fig. 4. The start time for this device is slightly longer than that of the previous one because of the application of reflector^37^. However, the output power reaches as high as 138 MW, which is far progressed compared to the previous model. The efficiency is also improved from 10.7% to 16.2% since the adoption of dual-reflector. The frequency is slightly shifted to 337.7 GHz and the spectrum is steady. As can be seen in Fig. 4(b), the distributions for *E*_z_ are compared along various observing lines. The observing lines are set at different longitudinal positions, namely the positions of the 8^th^, 12^th^, 16^th^, 20^th^ and 22^th^, respectively. The lines are placed along the radial directions from *r* = 0 to the surface of SWS. It is clear in Fig. 4(b) that the TM_01_ mode, locating at the surface of SWS, surely has the highest field strength. Besides TM_01_ mode, a few high order modes also exist in the SWS. Nevertheless, as the electron travels at the position close to the SWS surface, it mainly interacts with the strong field of TM_01_ mode, ensuring the interaction efficiency.

At the same time, the structure of dual-reflector is helpful in minimizing the negative going power flux and in enlarging the pre-modulation on beam. Based on the PIC simulations on the devices with and without the reflector, the performance is compared in Fig. 4(c). Obviously, the power flowing towards the diode area is distinct and serious when there is no reflector. The negative going power would be harmful to the device’s operation, affecting the cathode or the isolator. While, by applying the structure of dual-reflector, the problem of power going back is solved and the power going positive is also enhanced in the SWS area. Meanwhile, the modulation on beam is also reinforced in the positions of reflectors, as shown in Fig. 4(c). The pre-modulation on the electron beam is valid in promoting the subsequent energy conversion process. Thus the proposal of dual-reflector is quite meaningful and useful in this oversized SWO.

**Figure 5:**
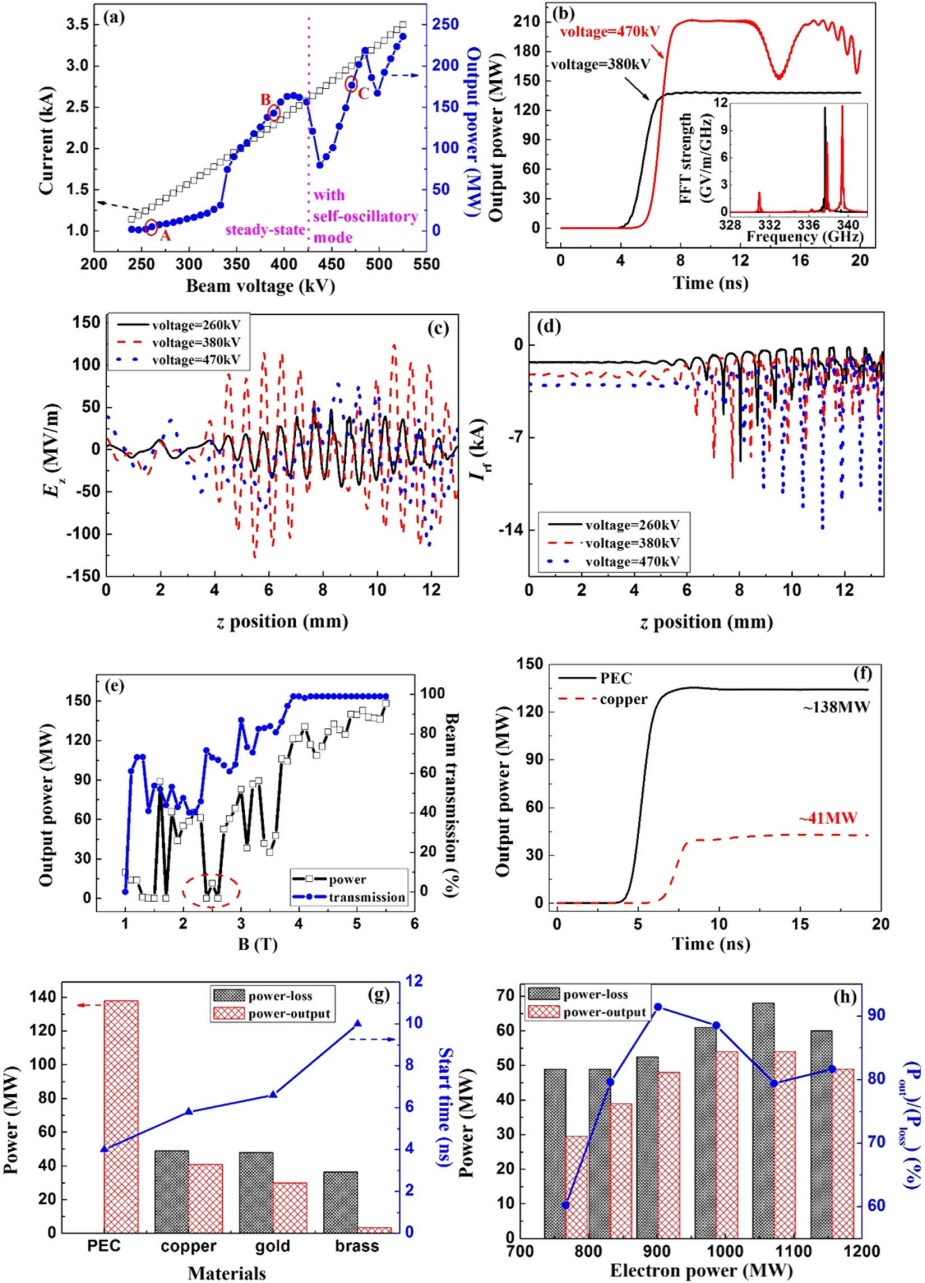
The influences of voltage, magnetic field and material on the output performances. (**a**) Output power vs. voltage; (**b**) comparison between the steady state and the self-oscillation state; (**c**) *E*_*z*_ vs. *z*; (**d**) Modulated current vs. *z*; (**e**) Output power vs. magnetic field; (**f**) Comparison of copper-SWS and PEC-SWS; (**g**) Output power and ohmic power on various materials; and (**h**) Output power and ohmic power under various voltage conditions for copper-SWS change of electron power in devices with different metals.

The influence of beam’s voltage on the output results are shown in Fig. 5(a). With the increase of beam’s voltage, the output power is rapidly improved. However, when the operating current exceeds a certain value, the device is not stable any longer due to the self-oscillatory mode. The detailed results for the steady state and the self-oscillatory state are compared in Fig. 5(b). Obviously, the device operates stably in the steady state and the frequency spectrum is pure. However, under the self-oscillatory state, multiple frequencies are excited simultaneously in the device. The peak value of the output power firstly exceeds the level for the stable state and then becomes periodically attenuated. According to Ginzburg’s non-stationary theory^54^, when the current significantly exceeds the starting current in BWO, the self-oscillatory mode is easily excited and the output signal would be periodically modulated. In this SWO, the phenomena is similar. In Fig. 5(a), the operating current is also compared with the increase of beam voltage. When the voltage is about 430 kV, the operating current is about 2.6 kA and from here on, the output results become unstable with the increase of operating current. The value of 2.6 kA is several times over the start current, which accords with the conclusion^54^. In order to further study the device’s status at different voltage conditions, three typical points, naming A, B and C in Fig. 5(a), are picked out to be analyzed in detail and the results are shown in Fig. 5(c,d). In case A, the voltage is quite low. Though the device locates in the designed status, the electric field is so weak that the modulation on beam is insufficient. Case B is just the optimum state. In this case, the voltages match the requirement for the operation near π point. Therefore, the beam is well modulated and the beam’s energy is gradually and fully extracted to the field. The situation in case C is associated with the self-oscillatory mode. The beam’s energy is firstly extracted to the beam and when it approaches the position of collector, the beam absorbs energy again so that the device appears to be unstable and in low efficiency. Consequently, in the optimum conditions, the device can operate well as expected and no self-oscillation would occur. In this stage, the practicable tuning range for the beam’s voltage is nearly 90 kV, which is meaningful for adjusting the device’s parameters in experiments.

The influence of magnetic field on the output power is presented in Fig. 5(e). Obviously, there is a special point around 2.5 T. The output results are sharply down compared to the other results. This accords well with the theoretical predictions on the cyclotron resonance absorbing^55^. In this device, when the Cerenkov synchronism and the cyclotron synchronism take place simultaneously at a certain magnetic field, the output power will be sharply reduced. Here, the cyclotron synchronism with both zero spatial harmonic of backward wave and −1^st^ harmonic of backward wave are taken into account^56,57^. The values of magnetic field can be estimated from ${B}_{1}=\frac{2m{v}_{0}}{e}{\gamma }_{0}\frac{\pi }{p}$ and ${B}_{2}=\frac{2m{v}_{0}}{e}{\gamma }_{0}(\frac{\pi }{p}-{k}_{0})$ for the two cyclotron absorbing conditions. Given the operation point is *k*_0_ = 0.95 π/*p*, the values of *B*_1_ and *B*_2_ are obtained as 47.6 T and 2.4 T. This agrees with the results in Fig. 5(c) that the minimal output power is obtained when the magnetic field is 2.5 T. As shown in Fig. 5(e), the output power grows with the increase of beam transmission in device. In this kind of relativistic devices, improving the transportation of beam is not only valid in promoting the efficiency but also necessary for the device’s safety. Therefore, the magnetic field in experiment is advised to be higher than 4.5 T.

As shown in Fig. 5(f), with the same working conditions of 382 kV and 2.2 kA, the output power drops from 138 MW for the PEC-structure to 41 W for the copper-structure, corresponding to the decrease of efficiency from 16.2% to 4.8%. Besides, the ohmic loss in the copper wall not only significantly decreases the output power, but also delays the start time for the oscillation. In experiments, the surface roughness during the micro-fabrication process is comparable to the skin depth of metal at the terahertz range. As a result, it will cause considerable power loss and deteriorate the steady situation eventually. What’s more, the electrical conductivity of metal strongly affects the performance of device^58-60^. As can be seen in Fig. 5(g), in the condition of metal with high conductivity, the level of output power is approaching the level of ohmic loss. In contrast, the situation in device with low conductivity is worse as the radiated power is far less than the loss power. Moreover, with the decrease of the conductivity of metal, the start time for device is obviously delayed. So in the device with low conductivity metal, such as stainless steel, the performance would be badly disserved. Oxygen-free copper is preferred in manufacturing the terahertz devices. The detailed results of copper-device with different voltage conditions are compared in Fig. 5(h). Clearly, as the increase of beam’s power, the activity of beam-wave interaction is enhancing and then the output power is increasing. Besides that, the ratio of output power with respect to the wasted power on metal in the cavity is also increasing and tends to be a constant around 80%.

To speed up the start time of the terahertz signal, we may inject the external small terahertz signal^61^ with our newly developed continuous-wave Y-band planar BWO^25^.

## Conclusion

The structure of oversized SWO is theoretically optimized to improve the efficiency. By analyzing the dispersion and coupling impedances for all modes, the problem of mode competition in this SWO is discussed. Through the approach of adopting a dual-reflector, the strength of TM_01_ mode in whole cavity is effectively enhanced compared to the other high order modes and accordingly the interaction efficiency is increased. The dual-reflector also acts well in preventing the power flux going back to the diode area. From the PIC simulations, the results show that the optimized device can operate stably and its efficiency is improved to 16.2% in the perfectly electrical conductivity condition. The output performance regarding the various conditions of beam voltage, guiding magnetic field, and the material of the SWS are also studied, which is helpful to recognize the status of device. Especially, when the device is made of copper, the output power is about 41 MW with the reduced efficiency of 4.8% due to the ohmic loss, illustrating that the lossy material seriously affects the performance of terahertz VEDs. Next, we will further optimize the parameters of the device structure and the driving electron beam^62^.
